# Phylogenetic analysis of zoonotic *Echinococcus granulosus sensu stricto* in humans and domestic animals from Armenia and Türkiye

**DOI:** 10.1017/S0031182026102170

**Published:** 2026-06

**Authors:** Urmas Saarma, Maris Pärn, Hripsime Atoyan, Teivi Laurimäe, Sami Simsek, Figen Celik, Sargis A. Aghayan, Hasmik Gevorgyan, Marion Wassermann, Thomas Romig, Adriano Casulli, Liina Anijalg

**Affiliations:** 1 Department of Zoology, Institute of Ecology and Earth Sciences, University of Tartu, Tartu, Estonia; 2 The American University of Armenia, Gerald and Patricia Turpanjian College of Health Sciences, Yerevan, Armenia; 3 Department of Parasitology, Faculty of Veterinary Medicine, University of Firat, Elazig, Türkiye; 4 Chair of Zoology, Yerevan State University, Yerevan, Armenia; 5 Laboratory of Molecular Parasitology, Scientific Center of Zoology and Hydroecology, Yerevan, Armenia; 6 Institute of Biology, Parasitology Unit, University of Hohenheim, Stuttgart, Germany; 7 European Union Reference Laboratory for Parasites (EURL-P). Department of Infectious Diseases, Istituto Superiore di Sanità, Rome, Italy; 8 WHO Collaborating Centre for the Epidemiology, Detection and Control of Cystic and Alveolar Echinococcosis (One Health). Department of Infectious Diseases, Istituto Superiore di Sanità, Rome, Italy

**Keywords:** cystic echinococcosis, genotype G1, genotype G3, mitochondrial genome, zoonoses, zoonotic

## Abstract

Cystic echinococcosis (CE) is a zoonotic disease of high public health and economic importance. The disease is caused by various tapeworm species belonging to the *Echinococcus granulosus sensu lato* species complex. Among these, *E. granulosus sensu stricto* (genotypes G1 and G3) is the most common cause of human CE worldwide. The aim of this study was to analyse the genetic diversity and phylogenetic relationships of G1 and G3 isolates from Armenia and Türkiye, where CE has long been recognized as a significant public health concern. A large portion of the mitochondrial genome (11 675 bp) was sequenced for 85 parasite samples: 47 from Türkiye and 38 from Armenia. Samples originated from humans, sheep and cattle. For a global phylogenetic analysis, sequences from previous studies were included, yielding 280 G1 sequences and 55 G3 sequences from various host species and countries worldwide. The main results of this work revealed (i) high genetic diversity of G1 in Armenia and Türkiye; (ii) high genetic diversity of G3 in Armenia, whereas only a few representatives of G3 were detected in Türkiye; (iii) the subdivision of isolates of G1 and G3 from Armenia and Türkiye into several haplogroups; (iv) the dispersion of G1 and G3 haplotypes from Armenia and Türkiye across different haplogroups in global phylogenetic networks; and (v) the diffusion routes of G3 isolates from Türkiye to Armenia, from Iran to Armenia and from Iran to Greece. These findings highlight the importance of mitogenome analysis for monitoring the spread of CE among animals and humans.

## Graphical abstract

**Figure:**
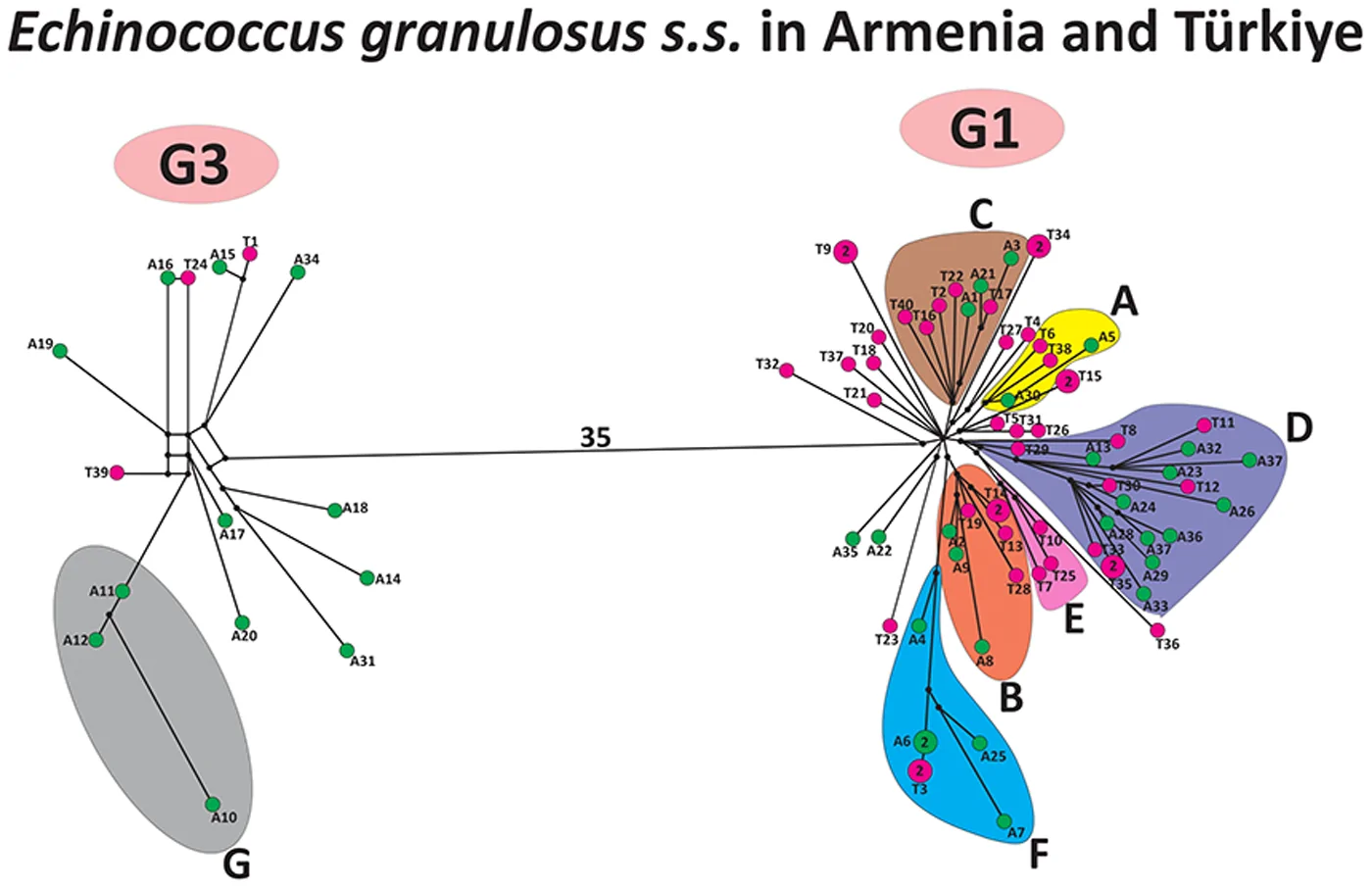

## Introduction

Cystic echinococcosis (CE) is a zoonotic disease, caused by tapeworm parasites of the *Echinococcus granulosus sensu lato* (*s.l.*) species complex. In this complex, the species *E. granulosus sensu stricto* (*s.s.*) is geographically the most widespread and of the highest zoonotic importance, being the most common cause of human CE worldwide. Wide-scale analysis of human CE data has revealed that >80% are due to *E. granulosus s.s.*, followed by genotypes G6/G7 (Alvarez Rojas et al., 2014; Casulli et al., 2022). The highly zoonotic *E. granulosus s.s.* primarily affects people living in rural areas and leading a pastoral lifestyle that provides a favourable environment for its life cycle (Deplazes et al., 2017; Romig et al., 2017; Thompson, 2017). Its main definitive host is the dog, whereas sheep, cattle and buffalo dominate among intermediate hosts. Wild animals are less involved, depending on the region (Romig et al., 2017; Romig and Wassermann, 2024). *Echinococcus granulosus s.s.* is also significant for food safety. A recent multicentre study from the MEmE project (https://onehealthejp.eu/projects/emerging-threats/jrp-meme; OneHealth EJP framework) by Umhang et al. (2025), involving 12 European countries, Tunisia and Pakistan, reported the presence of *E. granulosus s.s.* eggs in lettuce and berries. Regarding lettuce, the frequency of occurrence of *E. granulosus s.s.* ranged from 0% to 6·5% among European countries, whereas in Pakistan and Tunisia, it was 4% and 12%, respectively. Among berries, the highest frequency of occurrence of *E. granulosus s.s.* was reported for blueberries from Pakistan (12%) and for strawberries from Tunisia (81·3%).

Phylogenetic studies of the genus *Echinococcus* have demonstrated that *E. granulosus s.s.* forms a monophyletic clade and is a sister species to *E. felidis* (e.g., Saarma et al., 2009; Knapp et al., 2011; Lymbery, 2017). The intraspecific phylogenetic analysis based on mtDNA has revealed that *E. granulosus s.s.* is divided into 2 major clades, one comprising representatives of genotype G1 and the other of G3 (Kinkar et al., 2017). These genotypes can be confidently distinguished from each other by sequencing a fragment of the mtDNA *nad5* gene (Kinkar et al., 2018a). Both genotypes are further divided into multiple subclades (Kinkar et al., 2018b,c). However, nuclear DNA analysis revealed no subdivision, suggesting ongoing gene flow between G1 and G3 (Kinkar et al., 2017). The genotype G1 (formerly the ‘sheep strain’) has a global distribution and can infect a wide range of animals and humans. The geographical distribution of G3 (formerly the ‘buffalo strain’) and the number of host species are, according to current knowledge, not as wide as those of G1 (Kinkar et al., 2018c). In recent years, sequencing a large portion of mitochondrial genomes has considerably improved our understanding of the phylogenetic relations of isolates of G1 and G3 from different regions worldwide (Kinkar et al., 2016, 2017, 2018a, 2018b, 2018c; Laurimäe et al., 2016; Zhao et al., 2022; Saarma et al., 2023; Guo et al., 2024). The complex phylogeographical structure emerging from these studies has suggested that much of the current distribution, especially of G1, has been shaped by animal trade. Long mtDNA sequences can be crucial for correct genotype identification (Laurimäe et al., 2018; Biedermann et al., 2025) and may help identify the origin of infection. For example, Kinkar et al. (2017) described a case involving a G1 haplotype from Finland that clustered together with several other haplotypes from Algeria. As the isolate was from an Algerian patient living in Finland, it is highly likely that the geographical origin of the infection was Algeria.

In Armenia, 752 surgical cases of CE were reported during 2008–2022, with an annual average incidence of 2·7 per 100 000 individuals. The majority of infected people (58%) were from rural communities (Manukyan et al., 2024). Molecular studies of the *E. granulosus s.l.* species complex in Armenia have been carried out since 2005, revealing *E. granulosus s.s.* as the dominant species in humans, sheep and cattle (Gevorgyan, 2006; Gevorgyan et al., 2006; Ebi et al., 2014). However, exact genotype identifications have been rare (Maksimov et al., 2020).

In Türkiye, according to the Social Security Administration, in 2008–2012, a total of 32 261 CE patients received treatment and 12 556 underwent surgical intervention (Ok et al., 2020). Other studies have confirmed that *E. granulosus s.s.* has dominated among human patients (Utuk et al., 2008; Ergin et al., 2010; Simsek et al., 2011b; Eryildiz and Şakru, 2012; Bakal et al., 2015; Orsten et al., 2018; Akkas et al., 2023; Celik et al., 2024; Yılmaz et al., 2025). Among domestic animals, *E. granulosus s.s.* appears most common in sheep, cattle and dogs (e.g., Vural et al., 2008; Borhani et al., 2021). Moreover, in a recent study, 7 of 13 cysts from goats were identified as *E. granulosus s.s.* (Selcuk et al., 2024). Celik et al. (2024) reported that out of 28 cattle samples, 26 were G1 and 2 were G3, and out of 31 sheep samples, 25 were G1 and 6 were G3. Of wild animals, *E. granulosus s.s.* has been reported in wild sheep (*Ovis gmelinii anatolica*) (Simsek and Eroksuz, 2009), wild boar (*Sus scrofa*) (Kesik et al., 2021) and wolf (*Canis lupus*) (Akyuz et al., 2024).

Although Armenia and Türkiye are neighbouring countries where CE has long been recognized as a significant public health concern, little is known about the genetic variation and phylogenetic relationships among *E. granulosus s.s.* isolates from these 2 countries. The aim of this study was to analyse the mitogenome diversity and phylogenetic relationships of G1 and G3 isolates from Armenia and Türkiye and reveal major phylogeographical migration routes in the region.

## Materials and methods

### Samples

**Figure 1.**
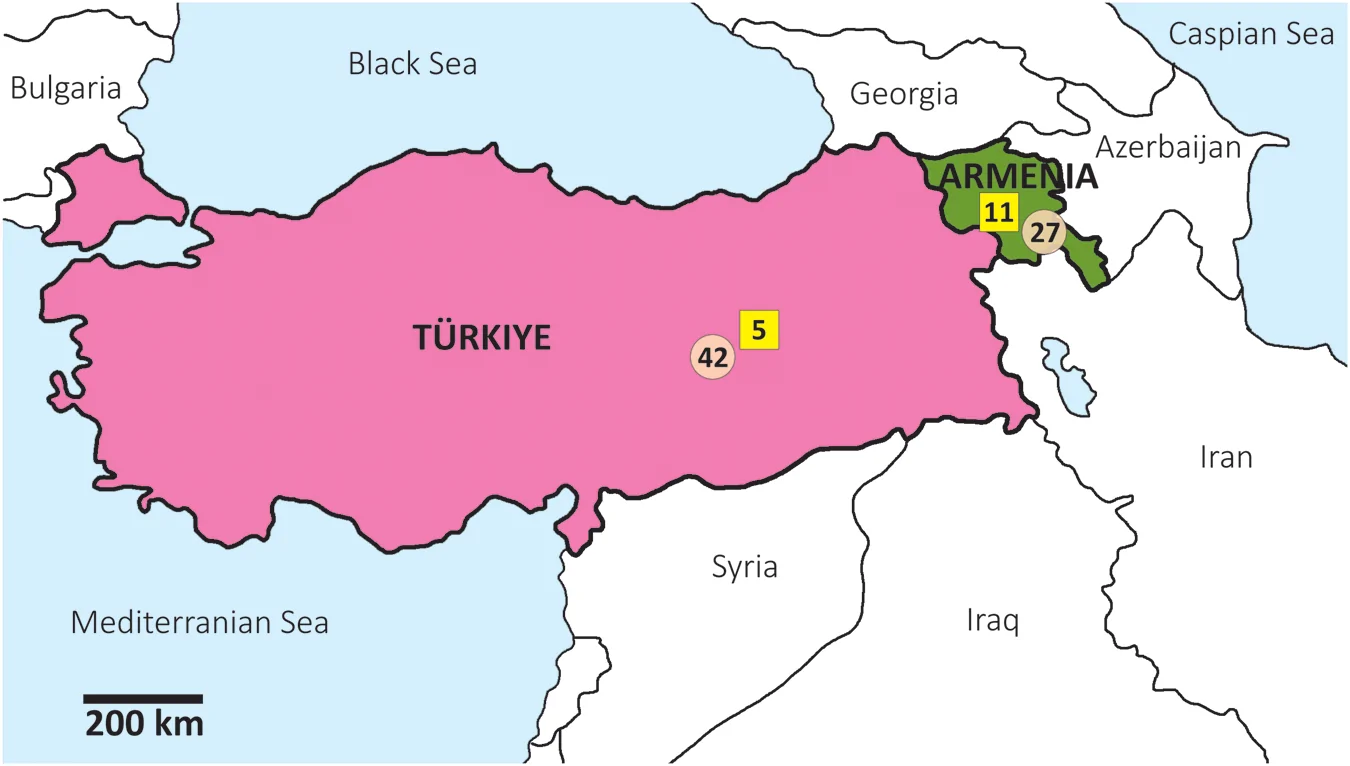
Geographical locations of *Echinococcus granulosus sensu stricto* isolates from Armenia and Türkiye. The number of genotype G1 samples is represented in circles, and those of G3 in rectangles. Figure 1 long description.

In this study, 85 parasite tissue samples (protoscoleces or cyst membranes) were analysed – 38 from Armenia and 47 from Türkiye (Figure 1; Table S1). From Armenia, 19 samples were from sheep, 8 from cattle and 11 from humans. From Türkiye, 23 samples were from sheep, 19 from cattle and 5 from humans.

### DNA purification, PCR and sequencing

DNA was purified from protoscoleces or cyst membranes using the High Pure PCR Template Preparation Kit (Roche Diagnostics, Mannheim, Germany), following the manufacturer’s protocols. For PCR amplification, 12 primer pairs were used, yielding overlapping DNA fragments (Table S2). Initially, to assess sample quality and determine the genotype, the primer pair E11f–E11r was used. For samples that yielded high-quality sequences, the remaining 11 loci were then PCR amplified and sequenced. While most primer sequences have been published earlier (Kinkar et al., 2016; Laurimäe et al., 2018), primers S7f and S11f are novel and were designed as additional sequencing primers. Note that primers E9f3–E9r3 reported in Laurimäe et al. (2018) fail to amplify the target region in *E. granulosus s.s.* due to a large non-coding region reported in Kinkar et al. (2019) and were therefore not used in this study.

PCR was performed in 20 µL comprising 1× BD Advantage 2 PCR buffer (BD Biosciences, Franklin Lakes, NJ, USA), 0·2 mM dNTP, 0·25 μM of each primer, 1 U of Advantage 2 Polymerase mix (BD Biosciences) and 10–100 ng of template DNA. Negative controls were always included. PCR cycling conditions were: 95 °C for 1 min, followed by 10 cycles of 95 °C for 20 s, 55 °C for 45 s (annealing temperature reduced by −0·5 °C per cycle) and 68 °C for 2 min; followed by 25 cycles of 95 °C for 20 s, 50 °C for 45 s, 68 °C for 2 min; and with a final extension at 68 °C for 3 min. Of the PCR product, 10 µL was examined on a 1% agarose gel in 1× TAE buffer, and for sequencing, 10 µL was purified with FastAP/Exonuclease I (both 1 unit; Thermo Fisher Scientific, Waltham, MA, USA) by incubating at 37 °C for 30 min and then inactivating the enzymes at 80 °C for 15 min. Sequencing was performed at the Core Facility at the Institute of Genomics (University of Tartu, Estonia), using the same set of primers as for the initial PCR, and 2 additional primers S7f and S11f (Table S2), to cover sequence gaps. Both forward and reverse strands were sequenced.

Sequences were assembled using CodonCode v8.0.2, and chromatograms were inspected visually and corrected where necessary. Sequences were manually edited and aligned in BioEdit v7.2.5 (Hall, 1999). All sequences were deposited in the GenBank database and are available under accession numbers PX908801-PX908869 (G1) and PZ091242-PZ091257 (G3) (Table S1).

### Phylogenetic analyses

To evaluate the phylogenetic position of the G1 and G3 haplotypes from Armenia and Türkiye in a global context, previously published sequences of G1 and G3 were retrieved from GenBank. For phylogenetic analyses, the following datasets were used:

(1) Dataset 1: ‘Armenia and Türkiye of this study.’ G1 and G3 samples from Armenia (*n* = 38) and Türkiye (*n* = 47) from this study, total *n* = 85;

(2) Dataset 2: ‘Global G1.’ G1 samples from Armenia (*n* = 26) and Türkiye (*n* = 43) of this study, along with 211 sequences of G1 from Kinkar et al. (2018b), total *n* = 280;

(3) Dataset 3: ‘Global G3.’ G3 samples from Armenia (*n* = 12) and Türkiye (*n* = 4) of this study, along with 39 G3 sequences from Kinkar et al. (2018c), total *n* = 55.

Alignment was performed with MAFFT online version (Katoh et al., 2019). Median-joining networks were constructed using the program Network v.10.1.00, with both indels and point mutations considered (Bandelt et al., 1999; http://www.fluxus-engineering.com, Fluxus Technology Ltd., 2004).

For Bayesian phylogenetic tree inference, the best-fit model of sequence evolution was estimated with PartitionFinder v2.1.1 (Guindon et al., 2010; Lanfear et al., 2012, 2016). Program BEAUti v1.8.4 was used to generate *xml* files used subsequently for BEAST 1.8.4 (Drummond et al., 2012) to infer the phylogenetic trees. The exponential growth coalescent prior and a strict molecular clock were applied, given the intraspecific nature of the data (Drummond and Bouckaert, 2015). The Markov Chain Monte Carlo chains were run for 10 million states, sampled every 1000 states, and 10% burn-in was discarded. Log files were analysed using the program Tracer v1.6 (Rambaut et al., 2014). Phylogenetic trees were produced in TreeAnnotator v1.8.4 and visualized in FigTree v.1.4.3 (Rambaut, 2014).

### Population indices

For the analysis of population indices, the same 3 datasets were used as described above for the phylogenetic analyses. The number of haplotypes, haplotype diversity, nucleotide diversity and Tajima’s *D* (Tajima, 1989) were calculated using DnaSP v6.12.03 (Rozas et al., 2017). Haplotype diversity is the probability that 2 randomly sampled haplotypes are different. Nucleotide diversity is the average number of nucleotide differences per site between 2 randomly chosen DNA sequences. Tajima’s *D* tests whether the observed pattern of polymorphism in a set of DNA sequences is consistent with a neutral model of evolution.

### Bayesian phylogeographical analysis

The Bayesian discrete phylogeographical approach was used to analyse phylogeographical diffusion (migration) directions (Lemey et al., 2009). This method allows for revealing ancestral locations from the provided set of samples and their locations and annotates the discrete location states to tree nodes. The analysis was performed essentially as described in Kinkar et al. (2018b). Three independent runs were performed, and highly significant routes ((Bayesian factor)BF > 1000) were displayed.

## Results

### Genotype identification and genotype ratios

Of the 38 isolates from Armenia, 26 were identified as *E. granulosus s.s.* genotype G1 (14 from sheep, 6 cattle, 6 humans) and 12 as genotype G3 (5 sheep, 2 cattle, 5 humans) (Table S1). Of the 47 samples from Türkiye, 43 were identified as G1 (21 sheep, 18 cattle, 4 humans) and 4 as G3 (2 sheep, 1 cattle, 1 human). Among the analysed Armenian samples, the overall G1:G3 ratio was close to 2:1 (26/12), whereas among samples from Türkiye, the ratio was nearly 11:1 (43/4). In Armenia, G1:G3 ratios for humans and animals were 6:5 and 3:1, respectively, whereas in Türkiye, the respective ratios were 4:1 and 13:1.

### Phylogenetic relations of G1 and G3 isolates from Armenia and Türkiye

**Figure 2.**
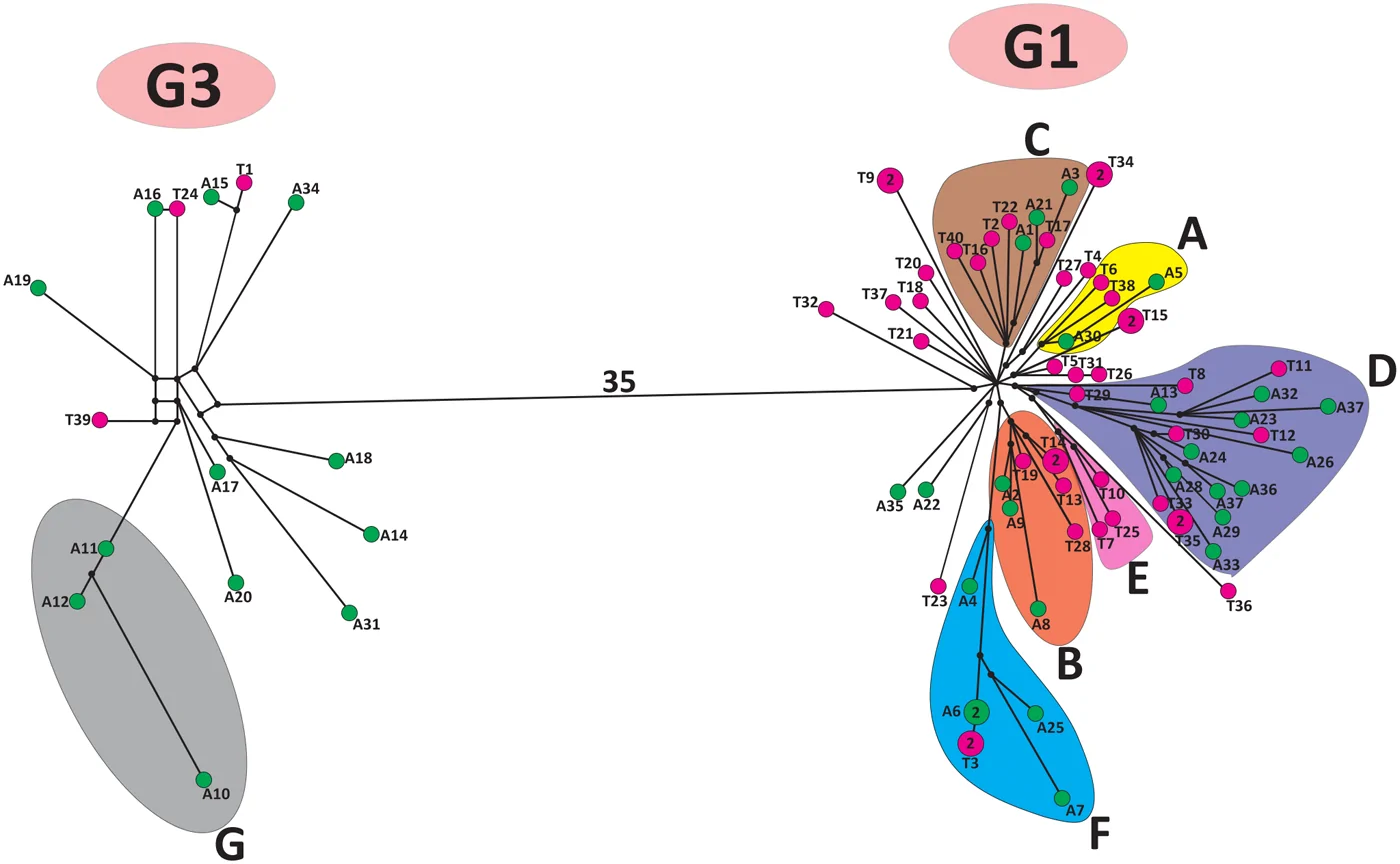
Median-joining network of *Echinococcus granulosus sensu stricto* G1 and G3 isolates from Armenia and Türkiye from this study (*n* = 85), based on 11 675 bp of mitochondrial genome sequences. The number of mutations between genotypes is depicted above the line. Haplotypes from Armenia are in green, and those from Türkiye are in purple (T: haplotypes of this study; TUR: haplotypes from previous studies). Haplogroups with very high support in the Bayesian phylogeny (see Figure 3) are circled and coloured differently. Small black dots are median vectors (i.e., Haplotypes not sampled or extinct). Figure 2 long description.

**Figure 3.**
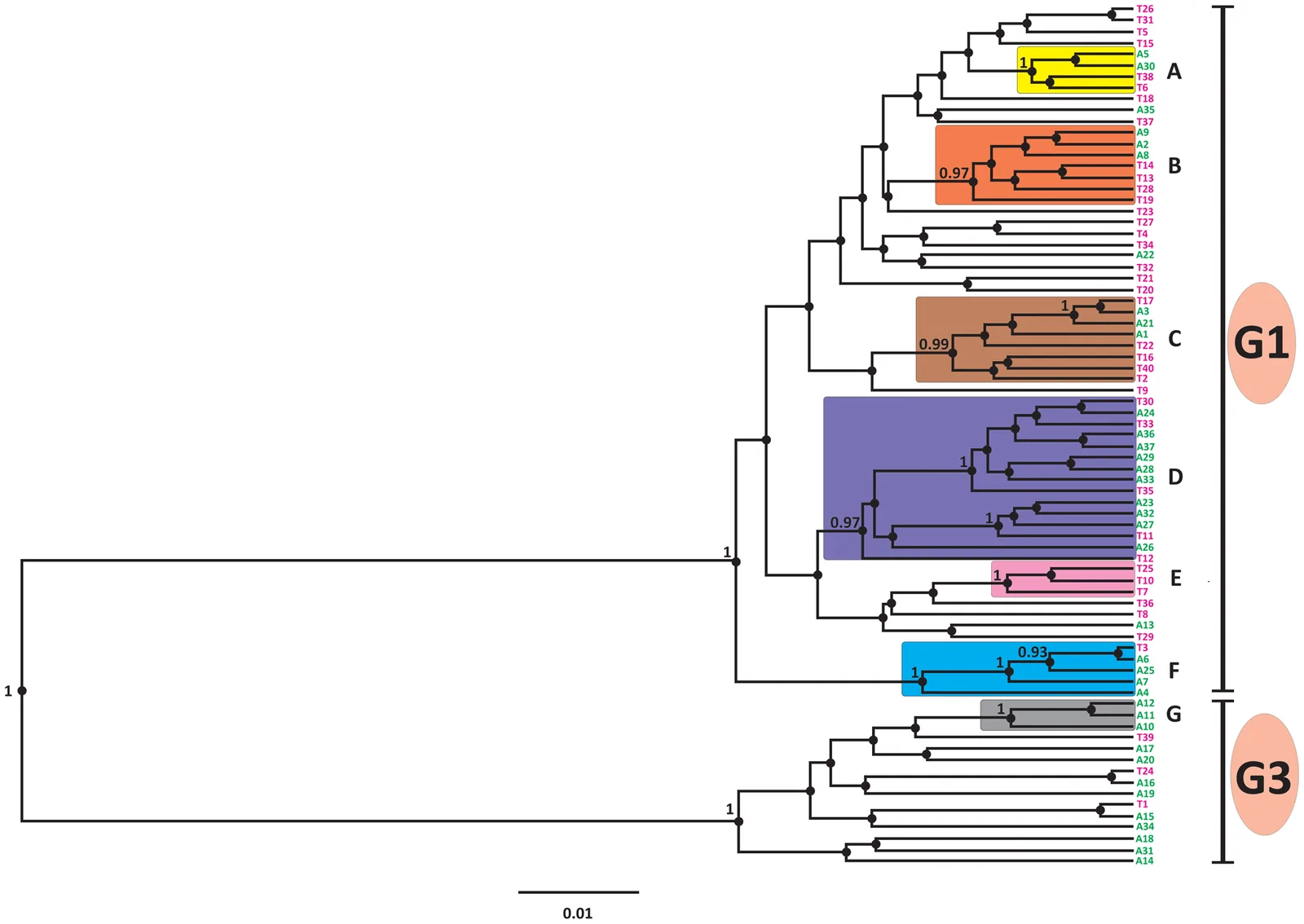
Bayesian phylogenetic tree of *Echinococcus granulosus sensu stricto* G1 and G3 isolates from Armenia and Türkiye, based on 11 675 bp of mitochondrial genome sequences (*n* = 85). Haplotypes from Armenia are in green, and those from Türkiye are in purple (T: haplotypes of this study; TUR: haplotypes from previous studies). Haplogroups with high posterior probability values (>0·95) are depicted in rectangles with different colours. Figure 3 long description.

Analysis with all 12 primer pairs (Table S2) yielded a 12 319 bp sequence alignment (note that the complete mitogenome of G1 exceeds 17 kb; Kinkar et al., 2019). The median-joining phylogenetic analysis, based on the alignment of 85 sequences from Armenia and Türkiye (Dataset 1), produced a network with 2 major clades, one comprising sequences of G1 and the other of G3 (Figure 2). The division into G1 and G3 received maximum posterior probability value (1·00) by the Bayesian phylogenetic inference (Figure 3). The Bayesian analysis revealed 6 highly supported subclades of G1 (A–F in Figure 3) and 1 of G3 (subclade G), with the posterior probability values 0·97 or higher. Most subclades included haplotypes from both Armenia and Türkiye, except for the G1 subclade E, which comprises solely isolates from Türkiye (*n* = 3), and the G3 subclade G, which included samples only from Armenia (*n* = 3). The genetic diversity of G1 and G3 was very high. The vast majority of analysed isolates had a unique mtDNA sequence (Figure 2).

### Diversity and neutrality indices

Samples with genotypes G1 and G3 from Armenia, and G1 from Türkiye exhibited high haplotype diversity values (Hd > 0·99), whereas G3 samples from Türkiye had a somewhat lower value (Hd = 0·83). The Tajima’s *D* neutrality indices were negative and statistically significant for G1 from Armenia and Türkiye, and G3 from Türkiye, suggesting rapid radiation (Table 1).

**Table 1.** Diversity and neutrality indices for *Echinococcus granulosus sensu stricto* genotypes G1 and G3 from Armenia and Türkiye, based on analysis of mitochondrial DNA (11 675 bp) Table 1 long description.

	Diversity				Neutrality
	*n*	Hn	Hd ± SD	π ± SD	Tajima’s *D*
Armenia					
G1	26	25	0·997 ± 0·001	0·00144 ± 0·00008	−1·916*
G3	12	12	1·000 ± 0·034	0·001161 ± 0·00014	−1·710
Türkiye					
G1	43	37	0·993 ± 0·006	0·00127 ± 0·00007	−2·425**
G3	4	3	0·833 ± 0·222	0·00111 ± 0·00029	−2·386^***^

*n*, number of samples; Hn, number of haplotypes; Hd, haplotype diversity; π, nucleotide diversity; SD, standard deviation.

* Significant *P*-value (*P* ≤ 0·05).

** Significant *P*-value (*P* ≤ 0·01).

*** Highly significant *P*-value (*P* ≤ 0·0001).

### Phylogenetic relations of G1 and G3 isolates from Armenia and Türkiye at a global scale, and migration routes

**Figure 4.**
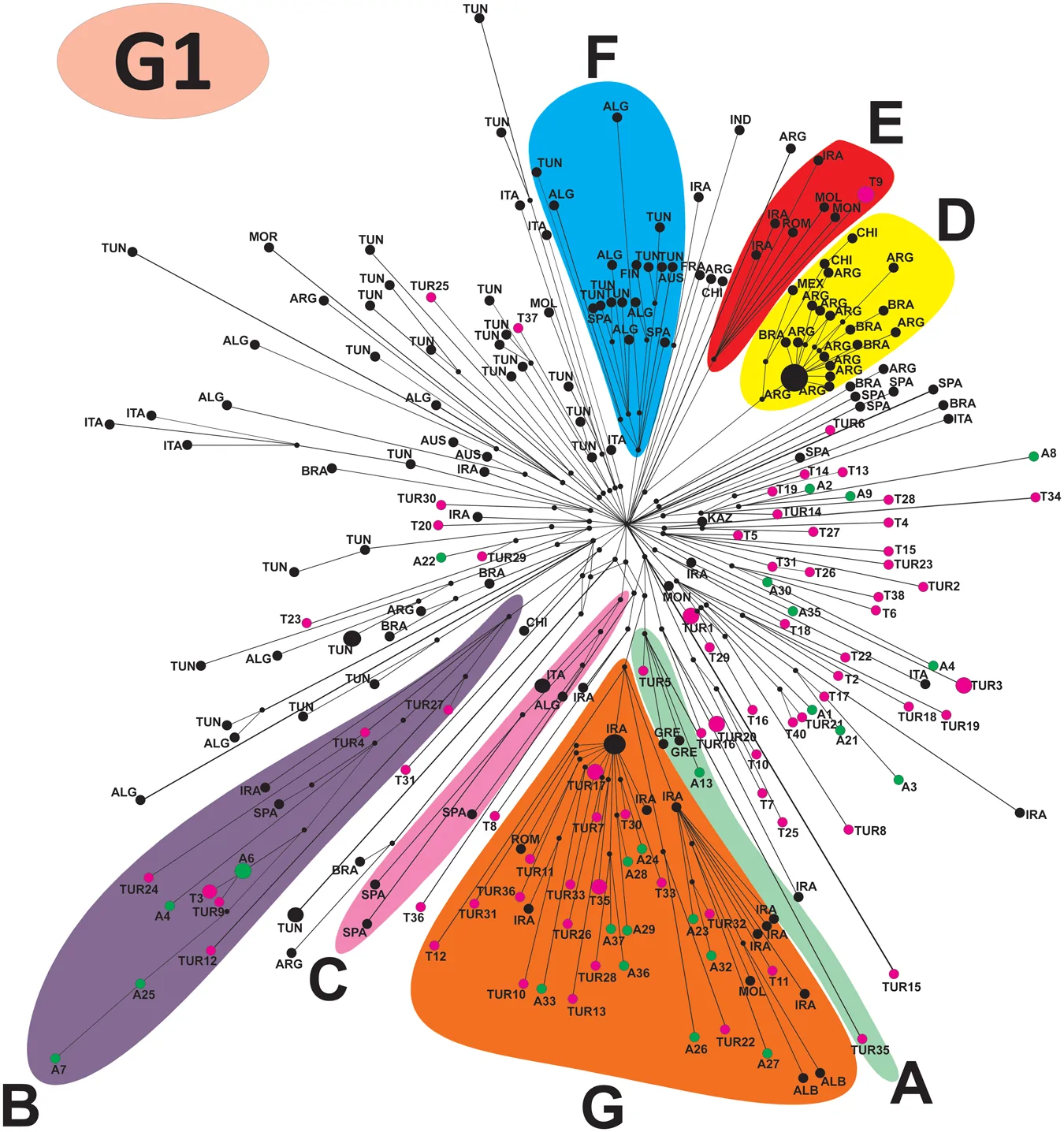
Global median-joining network of *Echinococcus granulosus sensu stricto* G1 isolates (total *n* = 280): from Armenia (*n* = 26) and Türkiye (*n* = 43) of this study, and 211 sequences of G1 from Kinkar et al. (2018b). Haplotypes from Armenia (A) are in green, and those from Türkiye are in purple (T: haplotypes of this study; TUR: haplotypes from previous studies). Figure 4 long description.

The global phylogenetic network of G1 based on Dataset 2 consisted of multiple haplogroups A–G (Figure 4), similar to that published in Kinkar et al. (2018b). The haplotypes from Armenia and Türkiye in this study remained closely related and were distributed across multiple haplogroups. A relatively large haplogroup G comprised haplotypes from both Armenia and Türkiye, as well as representatives from geographically proximate Iran. Haplotypes of both Armenia and Türkiye were also present in haplogroups A and B, whereas a single haplotype from Türkiye clustered into haplogroup E, which also included haplotypes from Iran, Romania, Moldova and Mongolia. These haplogroups also had representatives originating from more distant countries, such as Albania, Romania, Greece, Moldova and Spain.

**Figure 5.**
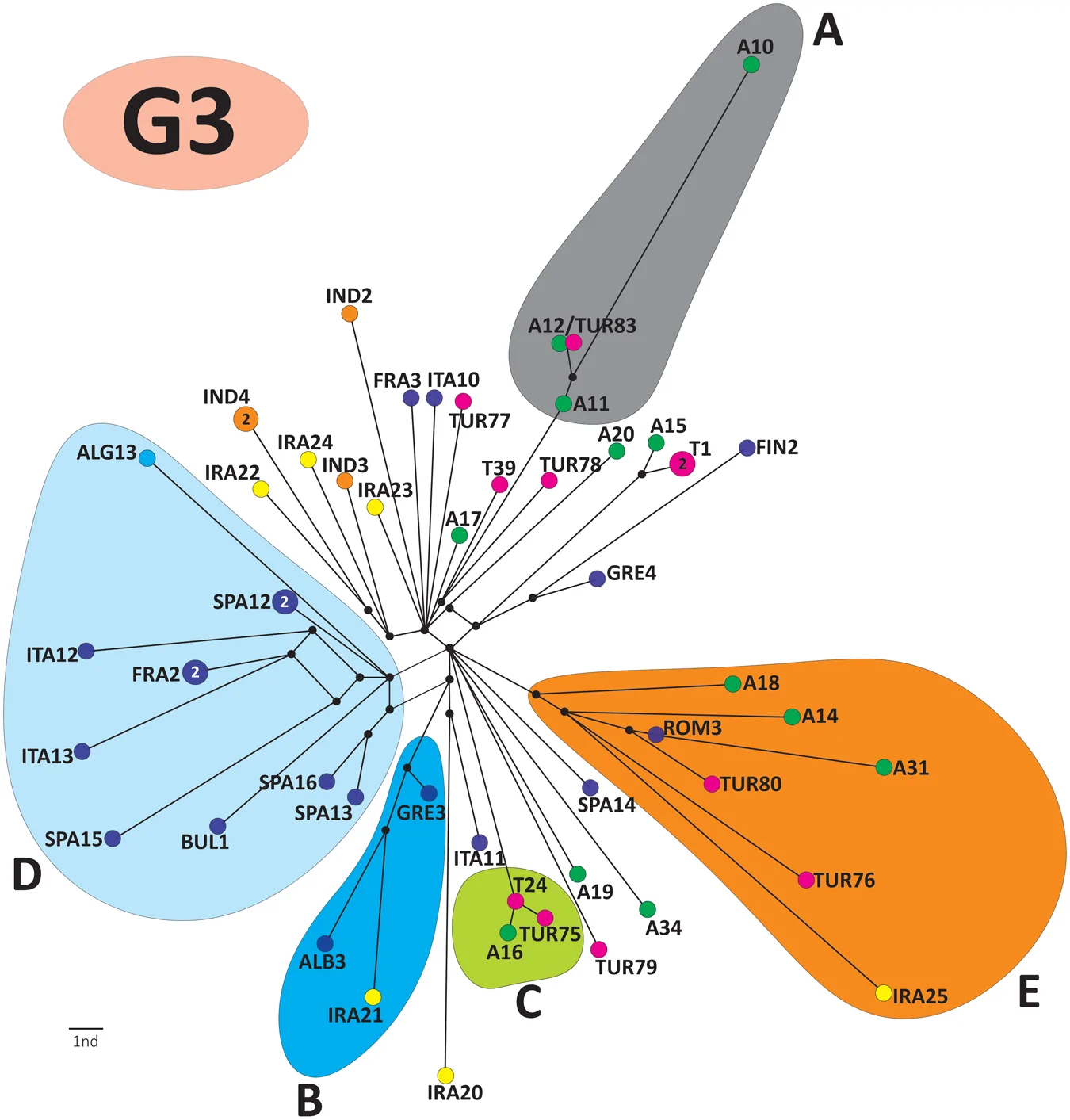
Global median-joining network of *Echinococcus granulosus sensu stricto* G3 isolates (total *n* = 55) from Armenia (*n* = 12) and Türkiye (*n* = 4) of this study, plus 39 G3 sequences from Kinkar et al. (2018c). The analysis is based on 11 675 bp of mitochondrial genome sequences. Haplotypes from Armenia are in green, and those from Türkiye are in purple (T: haplotypes of this study; TUR: haplotypes from previous studies). Figure 5 long description.

**Figure 6.**
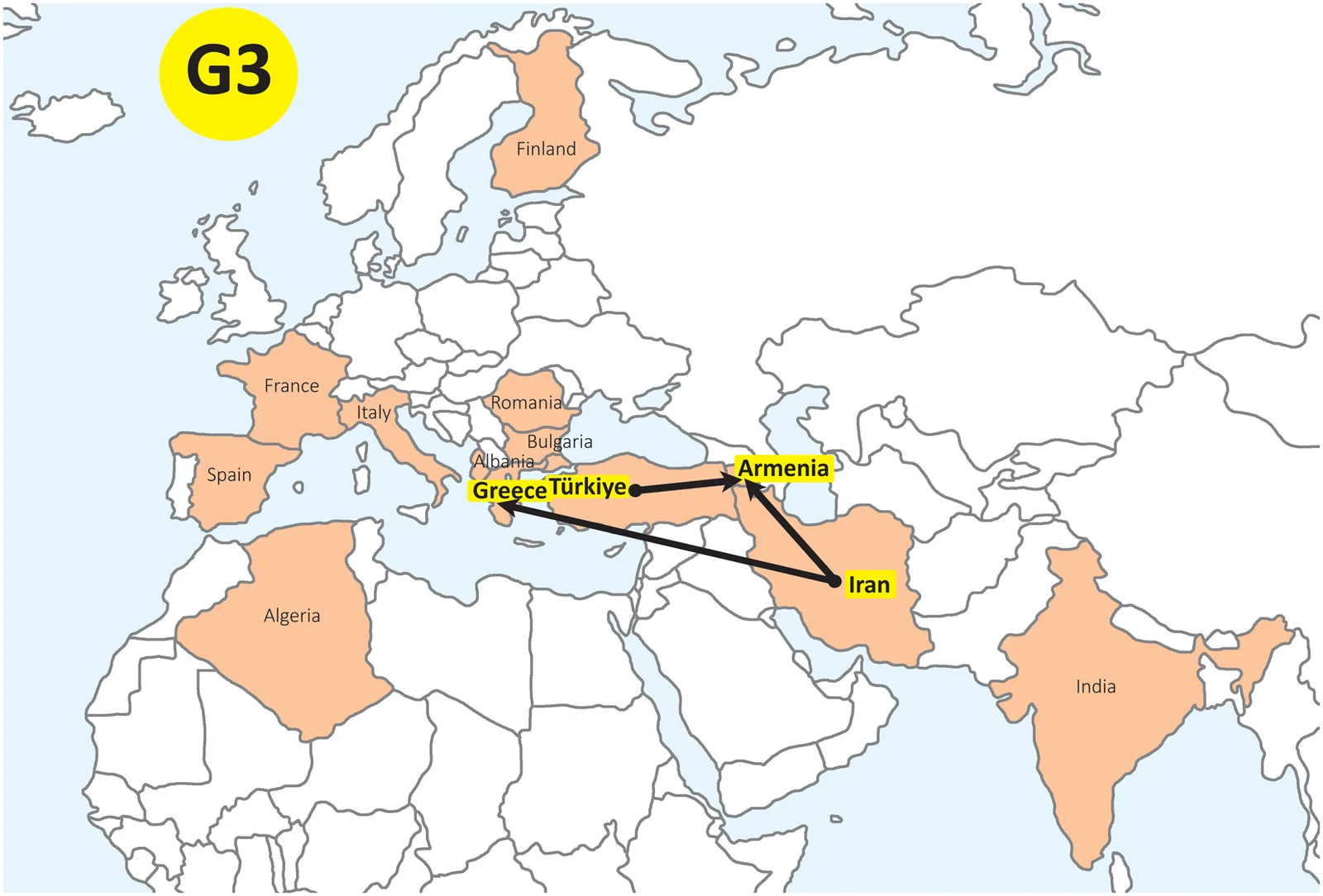
Major migration routes for *Echinococcus granulosus sensu stricto* genotype G3 isolates (*n* = 55), inferred from the Bayesian phylogeographical analysis. Black lines represent highly significant routes (BF > 1000). The analysis is based on samples from Armenia (*n* = 12) and Türkiye (*n* = 4) of this study, and 39 additional G3 sequences from 12 countries (Kinkar et al., 2018b). Figure 6 long description.

The global phylogenetic network of G3 (Dataset 3) comprised 5 well-supported haplogroups (A–E; Figure 5; see also Kinkar et al., 2018c). Representatives of G3 from Armenia and Türkiye clustered into haplogroups A, C and E with representatives of other countries. However, haplogroups A and C contained isolates only from Armenia and Türkiye. The Bayesian phylogeographical analysis revealed several highly supported (BF > 1000) migration routes of G3 from Iran to Armenia, and from Türkiye to Armenia (Figure 6).

## Discussion

*Echinococcus granulosus s.s.* (G1/G3) appears to be the most abundant *Echinococcus* species infecting humans in Armenia and Türkiye. In this study, G1 was identified in 6 humans from Armenia and 4 from Türkiye, whereas G3 was found in 5 human patients in Armenia and 1 in Türkiye. In earlier studies involving human samples from Armenia and Türkiye, *E. granulosus s.s.* also prevailed (Gevorgyan et al., 2006; Šnábel et al., 2009; Orsten et al., 2018; Bakal et al., 2021; Hamamci et al., 2023).

Note that based on the human samples analysed in this study, the G1:G3 ratio in Armenia was almost equal (6:5), suggesting that in terms of capability to infect humans, both G1 and G3 can be considered of high zoonotic importance. Although the ratio was 4:1 in favour of G1 in Türkiye, it seems to reflect the overall predominance of G1 in Türkiye, rather than the higher zoonotic potential of G1.

The highly different ratios of G1:G3 observed among all samples (humans and animals combined) analysed in this study from Armenia (2:1; 26/12) and Türkiye (11:1; 43/4) may indicate different historical migratory scenarios for G1 and G3. However, among samples published from Türkiye in earlier studies (Kinkar et al., 2018b, c), the ratio of G1:G3 was lower (5:1; 42/8). Combining the numbers of the current and earlier studies yielded the total ratio of G1:G3 in Türkiye 7:1 (85/12). Thus, the ratio in Türkiye is considerably more in favour of G1 than in Armenia. Interestingly, the Bayesian phylogeographical analysis revealed diffusion (migration) routes of G3 from Iran to Armenia, and from Türkiye to Armenia (Figure 6). In the present study, the G1:G3 ratio for the samples published previously from Iran was in favour of G1 by 4:1 (Kinkar et al., 2018b, c), which is somewhat higher than in Armenia (2:1), but not as high as in Türkiye (7:1). This suggests that Iran may have contributed more to the Armenian parasite population. Türkiye and Iran were part of an ancient domestication centre for ruminants (sheep, cattle and goats). Signs of domestication appeared around 12 000–11 000 years ago in the Fertile Crescent, which includes parts of contemporary Iraq, Iran, Syria and Türkiye (Zeder, 2025). Differences in the G1:G3 ratio could, at least partially, reflect the domestication and subsequent spread of ruminants infected with *E. granulosus s.s*. However, it is difficult to assess whether the differences in G1:G3 ratios can be attributed to historical or more recent processes, and to what extent to sampling. More samples and wider geographical coverage in this region are required to resolve this question.

Several haplogroups of G1 and G3, which all include haplotypes from both Armenia and Türkiye, also comprise haplotypes from other countries, such as Iran, Albania, Romania, Greece, Moldova and Spain (Figures 4 and 5). This close phylogenetic relationship is likely due to animal transportation/trade among countries of the former Ottoman Empire, which controlled much of West Asia, Southeast and Central Europe, and North Africa from the 14th to the early 20th centuries. The main migration routes of G3, as revealed in the Bayesian phylogeographical analysis, support the transportation/trade, as the highly supported diffusion routes were found from Türkiye to Armenia, from Iran to Armenia and from Iran to Greece. Thus, Iran and Türkiye appear to be the main G3 ‘donor’ countries for Armenia. However, we would like to emphasize that Bayesian phylogeographical analysis is sensitive to sampling and the results should be interpreted with caution. Therefore, the established diffusion routes, although highly supported, can be regarded as a hypothesis.

Genetic data is crucial for targeted surveillance and control efforts of *E. granulosus s.l*. Although short mtDNA sequences are often sufficient to identify the species and genotype of the parasite causing CE, long mitogenome sequences can provide more detailed information on the genetic variation of parasites, their phylogenetic relationships and migratory patterns in the region. As more such data accumulate and become available, the more potent the analytical power will grow. Moreover, the registration of CE and AE cases by national health systems should be improved in the future, as not all human cases are reported (Casulli et al., 2023) and a significant number of cases are merely reported as echinococcosis rather than identified as either CE or AE (Casulli et al., 2026).

As a future direction, analysing both the mitogenome and whole-genome nuclear data *E. granulosus s.s.* could provide crucial insights into genomic variation, population structure, selective pressures and potential drug targets across hosts and geographic regions, aided by the availability of high-quality, chromosome-scale reference genomes (Korhonen et al., 2022).

## Supporting information

10.1017/S0031182026102170.sm001 Saarma et al. supplementary material 1 Saarma et al. supplementary material

10.1017/S0031182026102170.sm002 Saarma et al. supplementary material 2 Saarma et al. supplementary material

## Data Availability

The 11 675 bp mtDNA sequences of the *E. granulosus s.s.* G1 and G3 isolates are deposited in GenBank under accession numbers: G1 (PX908801-PX908869); G3 (PZ091242-PZ091257).

## Supplementary material

The supplementary material for this article can be found at https://doi.org/10.1017/S0031182026102170.

## Figure 1 Long description

The map displays Türkiye and Armenia, highlighting the geographical locations of isolates. Türkiye is marked in pink and Armenia in green. Genotype G1 samples are represented by circles, with 42 samples in Türkiye and 11 in Armenia. Genotype G3 samples are represented by rectangles, with 5 samples in Türkiye and 27 in Armenia. The map includes surrounding regions such as the Black Sea, Mediterranean Sea and neighboring countries like Georgia, Azerbaijan, Iran, Syria and Iraq. A scale bar indicates a distance of 200 km.

Navigate back to Figure 1.

## Figure 2 Long description

The diagram represents a median-joining network of G1 and G3 isolates from Armenia and Türkiye, based on mitochondrial genome sequences. It features two main clusters labeled G1 and G3. The G3 cluster includes nodes representing haplotypes, connected by lines indicating mutational steps. A shaded region labeled G encompasses several nodes. The G1 cluster contains multiple subgroups labeled A, B, C, D, E and F, each enclosed in different shaded regions. Nodes within these subgroups represent haplotypes, connected by lines showing mutational relationships. A long line labeled 35 connects the G1 and G3 clusters, indicating the number of mutations between them. Small black dots represent median vectors, indicating unsampled or extinct haplotypes. The diagram does not use axes or units, focusing instead on genetic relationships and subgroup classifications.

Navigate back to Figure 2.

## Figure 3 Long description

The diagram is a Bayesian phylogenetic tree with a vertical orientation, depicting the evolutionary relationships of G1 and G3 isolates. The tree is divided into two main clades labeled G1 and G3, with G1 on the top and G3 on the bottom. Each clade is further divided into subclades labeled A to G. Subclades A, B, C, D and F are part of G1, while subclade G belongs to G3. The branches are horizontal and connect various nodes, representing evolutionary events. Haplotypes from Armenia are marked in green and those from Türkiye are marked in purple. Posterior probability values are indicated on the branches, with values greater than 0.95 highlighted within colored rectangles. The scale bar at the bottom represents genetic distance, marked as 0.01. The tree shows high genetic diversity among the isolates, with unique mitochondrial DNA sequences for most. The division into G1 and G3 clades is supported by a maximum posterior probability value of 1.00.

Navigate back to Figure 3.

## Figure 4 Long description

”The diagram is labeled G1 in the upper left corner. It displays a median-joining haplotype network of G1 isolates. The network contains multiple nodes connected by lines. Node size varies, with larger nodes indicating higher haplotype frequency or sample count. Lines connecting nodes represent mutational steps and hash marks on connecting lines indicate the number of mutational steps between haplotypes. Seven distinct haplogroups are visible, labeled A, B, C, D, E, F and G. Each haplogroup is enclosed in a shaded region. Haplogroup G is the largest cluster and occupies the lower central area of the network. It contains the greatest number of nodes, including haplotypes labeled with country codes such as ARM for Armenia, TUR for Turkiye and IRN for Iran. Haplogroup A is located in the lower right area and contains nodes labeled with multiple country codes. Haplogroup B is located in the left area and contains a smaller number of nodes. Haplogroup C is a narrow elongated cluster positioned between haplogroups B and G. Haplogroup D is located in the upper right and contains a dense grouping of nodes. Haplogroup E is adjacent to haplogroup D in the upper right area and includes nodes from multiple countries. Haplogroup F is positioned in the upper central.

Navigate back to Figure 4.

## Figure 5 Long description

The diagram is a phylogenetic network of G3 isolates, displaying five haplogroups labeled A to E. The network is centered with branches radiating outward to each haplogroup. Haplogroup A is shaded in gray and includes nodes such as A10, A11 and A12, with connections to TUR83 and other nodes. Haplogroup B is shaded in blue, containing nodes like ALG13, ITA12 and IRA20. Haplogroup C is shaded in green, featuring nodes TUR75 and A16. Haplogroup D is shaded in light blue, with nodes such as SPA16 and ITA19. Haplogroup E is shaded in orange, including nodes A18, ROM13 and IRA25. Each haplogroup is connected by lines indicating genetic relationships, with nodes marked by colored circles representing different geographic origins. The network visually represents the genetic diversity and relationships among the G3 isolates.

Navigate back to Figure 5.

## Figure 6 Long description

A thematic regional map centered on Europe, the Mediterranean and western Asia, with north at the top. Text label: “G3” appears in a circular marker near the upper left. Highlighted areas: Several countries are filled to stand out from surrounding countries. These include Finland, France, Spain, Italy, Romania, Greece, Türkiye, Armenia, Iran, Algeria and India. Country labels visible on the map: Finland, France, Spain, Italy, Romania, Greece, Türkiye, Armenia, Iran, Algeria, India. Route symbols: Thick black lines connect labeled locations. The lines originate from the area labeled “Türkiye” and from the area labeled “Iran” and converge toward the area labeled “Armenia.” The lines are drawn with arrowheads indicating direction toward Armenia. Main connections shown: - Türkiye to Armenia. - Iran to Armenia.

Navigate back to Figure 6.

## Table 1 Long description

The table reports sample size, number of haplotypes, haplotype diversity with standard deviation, nucleotide diversity with standard deviation, and Tajima’s D for genotypes G1 and G3 in Armenia and Türkiye using mitochondrial DNA sequence data. Armenia G1 has 26 samples and 25 haplotypes with haplotype diversity 0.997 and nucleotide diversity 0.00144; Tajima’s D is negative at about minus 1.916. Armenia G3 has 12 samples and 12 haplotypes with haplotype diversity 1.000 and nucleotide diversity 0.001161; Tajima’s D is about minus 1.710. Türkiye G1 has 43 samples and 37 haplotypes with haplotype diversity 0.993 and nucleotide diversity 0.00127; Tajima’s D is about minus 2.425 and is marked as statistically significant. Türkiye G3 has 4 samples and 3 haplotypes with haplotype diversity 0.833 and nucleotide diversity 0.00111; Tajima’s D is about minus 2.386 and is marked as highly significant. Overall, haplotype diversity is very high in Armenia for both genotypes and in Türkiye for G1, but notably lower for Türkiye G3, and all Tajima’s D values are negative, with the most negative values in Türkiye. Comparisons involving Türkiye G3 should be interpreted cautiously because the sample size is very small and the uncertainty is large.

Navigate back to Table 1.

## References

[ref1] Akkaş Ö, Özgökçe M, Aydemir S, Dündar İ and Ekici A (2023) Genotyping of *Echinococcus granulosus* human isolates obtained from the PUNCTURE, ASPIRATION, INJECTION, and reAspiration (PAIR) process in Türkiye. *Iranian Journal of Parasitology* 18, 427–434. 10.18502/ijpa.v18i4.14242. PMC10758082 38169642

[ref2] Akyuz M, Kirman R, Guven E, Balkaya I and Avcioglu H (2024) *Echinococcus granulosus sensu stricto* and *Echinococcus multilocularis* in a gray wolf (*Canis lupus*) in Turkey: Further evidence for increased risk of alveolar echinococcosis in urban areas. *Acta Parasitologica* 69, 1319–1323. 10.1007/s11686-024-00842-x. PMC11182862 38662075

[ref3] Alvarez Rojas CA, Romig T and Lightowlers MW (2014) *Echinococcus granulosus sensu lato* genotypes infecting humans - review of current knowledge. *International Journal for Parasitology* 44, 9–18. 10.1016/j.ijpara.2013.08.008. 24269720

[ref4] Bakal U, Celik F, Simsek S, Kesik HK and Kilinc SG (2021) Genetic diversity and haplotypes of paediatric hydatid cyst isolates and first occurrence of *E. canadensis* (G6/G7) in paediatric cases in Turkey. *Parasitology* 148, 1482–1489. 10.1017/s0031182021001098. PMC11010141 34187611

[ref5] Bakal U, Simsek S and Kazez A (2015) Surgical and molecular evaluation of pediatric hydatid cyst cases in eastern Turkey. *Korean Journal of Parasitology* 53, 785–788. 10.3347/kjp.2015.53.6.785. PMC4725236 26797450

[ref6] Bandelt HJ, Forster P and Rohl A (1999) Median-joining networks for inferring intraspecific phylogenies. *Molecular Biology and Evolution* 16, 37–48. 10.1093/oxfordjournals.molbev.a026036 10331250

[ref7] Biedermann A, Laurimäe T, Anijalg L, Kamenetzky L, Soriano SV, Pierangeli N, Lazzarini LE, Umhang G, Bold B, Bayasgalan C, Karamon J, Samorek-pieórg M, Simsek S, Celik F, Harandi MF, Nasibi S, Mehmood N, Chihai O, Casulli A and Saarma U (2025) Zoonotic *Echinococcus granulosus sensu lato* genotypes G6 and G7: New insights from the global mitogenome analysis. *International Journal for Parasitology* 55, 569–579. 10.1016/j.ijpara.2025.04.014. 40250726

[ref8] Borhani M, Fathi S, Darabi E, Jalousian F, Simsek S, Ahmed H, Kesik HK, Hosseini SH, Romig T, Harandi MF and Mobedi I (2021) Echinococcoses in Iran, Turkey, and Pakistan: Old diseases in the new millennium. *Clinical Microbiology Reviews* 34, e0029020. 10.1128/cmr.00290-20. PMC8262809 34076492

[ref9] Casulli A, Abela B, Petrone D, Soba B, Dezsényi B, Karamon J, Millon L, Saarma U, Antolová D, Chappuis F, Gloor S, Stoeckle M, Müllhaupt B, Beck R, Lagler H, Lötsch F, Auer H, Hayette M-P, Kolářová L, Laivacuma S, Šarkūnas M, Sokolovas V, Marcinkutė A, Troell K, Deibel A, Jokelainen P, Sulima M, Krankowska D, Roman S, Joliat G-R, Halkic N, Bresson-hadni S, Bednarek J, Załęski A, Paul M, Yaqub S, Jensenius M, van der Giessen J, Nabarro L, Chiodini P, Demonmerot F, Knapp J, Grüner B, Kern P, Peters L, Santolamazza F and Santoro A (2026) Unveiling the incidences and trends of alveolar echinococcosis in Europe: A systematic review from the KNOW-PATH project. *The Lancet Infectious Diseases* 26, e49–61. 10.1016/S1473-3099(25)00283-X. 40578380

[ref10] Casulli A, Abela-ridder B, Petrone D, Fabiani M, Bobić B, Carmena D, Šoba B, Zerem E, Gargaté MJ, Kuzmanovska G, Calomfirescu C, Rainova I, Sotiraki S, Lungu V, Dezsényi B, Ortiz ZH, Karamon J, Maksimov P, Oksanen A, Millon L, Sviben M, Shkjezi R, Gjoni V, Akshija I, Saarma U, Torgerson P, Snabel V, Antolova D, Muhovic D, Besim H, Chereau F, García MB, Chappuis F, Gloor S, Stoeckle M, Müllhaupt B, Manno V, Santoro A and Santolamazza F (2023) Unveiling the incidences and trends of the neglected zoonosis cystic echinococcosis in Europe: A systematic review from the MEmE project. *The Lancet Infectious Diseases* 23, e95–e107. 10.1016/S1473-3099(22)00638-7. 36427513

[ref11] Casulli A, Massolo A, Saarma U, Umhang G, Santolamazza F and Santoro A (2022) Species and genotypes belonging to *Echinococcus granulosus sensu lato* complex causing human cystic echinococcosis in Europe (2000–2021): A systematic review. *Parasites & Vectors* 15, 109. 10.1186/s13071-022-05197-8. PMC8962544 35346335

[ref12] Celik F, Selcuk MA, Kilinc SG, Kesik HK, Ahmed H, Wang Y, Simsek S and Cao J (2024) Molecular discrimination of G1 and G3 genotypes of Echinococcus granulosus sensu stricto obtained from human, cattle, and sheep using the mitochondrial NADH dehydrogenase subunit 5 marker. *Acta Tropica* 252, 107124. 10.1016/j.actatropica.2024.107124. 38262573

[ref13] Deplazes P, Rinaldi L, Alvarez Rojas CA, Torgerson PR, Harandi MF, Romig T, Antolova D, Schurer JM, Lahmar S, Cringoli G, Magambo J, Thompson RCA and Jenkins EJ (2017) Global distribution of alveolar and cystic echinococcosis. *Advances in Parasitology* 95, 315–493. 10.1016/bs.apar.2016.11.001. 28131365

[ref14] Drummond AJ and Bouckaert RR (2015) *Bayesian Evolutionary Analysis with BEAST*. Cambridge, United Kingdom: Cambridge University Press.

[ref15] Drummond AJ, Suchard MA, Xie D and Rambaut A (2012) Bayesian phylogenetics with BEAUti and the BEAST 1.7. *Molecular Biology and Evolution* 29, 1969–1973. 10.1093/molbev/mss075. PMC3408070 22367748

[ref16] Ebi D, Gevorgyan H, Wassermann M and Romig T (2014) Genetic diversity of *Echinococcus granulosus sensu stricto* in Armenia. In Proceedings: Proceedings of the International Symposium “Innovation for the Management of Echinococcosis”. *Journal of Parasite* 21, 40.

[ref17] Ergin S, Saribas S, Yuksel P, Zengin K, Midilli K, Adas G, Arikan S, Aslan M, Uysal H, Caliskan R, Oner A, Kucukbasmaci O, Kaygusuz A, Torun MM and Kocazeybek B (2010) Genotypic characterisation of *Echinococcus granulosus* isolated from human in Turkey. *African Journal of Microbiology Research* 4, 551–555. 10.5897/AJMR.9000344.

[ref18] Eryildiz C and Şakru N (2012) Molecular characterization of human and animal isolates of Echinococcus granulosus in the Thrace Region, Turkey. *Balkan Medica Journal* 29, 261–267. 10.5152/balkanmedj.2012.072. PMC4115829 25207011

[ref19] Gevorgyan H (2006) Distribution and biological aspects of cystic echinococcosis (Echinococcus granulosus, Batsch, 1786) in Armenia. PhD thesis, Institute of Zoology NAS RA, Yerevan, Armenia, 119 pp.

[ref20] Gevorgyan H, Dinkel A, Mackenstedt U and Romig T (2006) Cystic echinococcosis in Armenia: Host species and Echinococcus genotypes. *Deutsche Gesellschaft Für Parasitologie*. Vol. 22, 147. Jahrestagung, Wien.

[ref21] Guindon S, Dufayard J-F, Lefort V, Anisimova M, Hordijk W and Gascuel O (2010) New algorithms and methods to estimate maximum-likelihood phylogenies: Assessing the performance of PhyML 3.0. *Systematic Biology* 59, 307–321. 10.1093/sysbio/syq010. 20525638

[ref22] Guo B, Cairen, Zhao L, Aimulajiang K, Tang W, Wu C, Yimingjiang M, Wu J, Mi R and Wen H (2024) First report of *Echinococcus granulosus* genotype 1 in a wild boar (*Sus scrofa*) from China. *Parasitology Research* 123, 236. 10.1007/s00436-024-08249-3. 38856927

[ref23] Hall TA (1999) BioEdit: A user-friendly biological sequence alignment editor and analysis program for windows 95/98/NT. *Nucleic Acids Symposium Series* 41, 95–98.

[ref24] Hamamci B, Açıkgöz G, Çetinkaya Ü, Kılıç E, Koçal S, Karaaslan K, Yetim TD and Yetim I (2023) Molecular genotyping of Echinococcus granulosus sensu stricto from human Echinococcal cysts in Hatay, Türkiye. *Experimental Parasitology* 245, 108454. 10.1016/j.exppara.2022.108454. 36587835

[ref25] Katoh K, Rozewicki J and Yamada KD (2019) MAFFT online service: Multiple sequence alignment, interactive sequence choice and visualization. *Briefings in Bioinformatics* 20, 1160–1166. 10.1093/bib/bbx108. PMC6781576 28968734

[ref26] Kesik HK, Çelik F, Kılınç SG, Karabulut B, Çevik A and Şimşek S (2021) Molecular characterization of hydatid cysts cases in a wild boar and mule in Turkey. *Turkish Journal of Parasitology* 45, 28–33. 10.4274/tpd.galenos.2020.6818. 33685065

[ref27] Kinkar L, Korhonen PK, Cai H, Gauci CG, Lightowlers MW, Saarma U, Jenkins DJ, Li J, Li J, Young ND and Gasser RB (2019) Long-read sequencing reveals a 4.4 kb tandem repeat region in the mitogenome of *Echinococcus granulosus* (sensu stricto) genotype G1. *Parasites & Vectors* 12, 238. 10.1186/s13071-019-3492-x. PMC6521400 31097022

[ref28] Kinkar L, Laurimäe T, Acosta-Jamett G, Andresiuk V, Balkaya I, Casulli A, Gasser RB, González LM, Haag KL, Zait H, Irshadullah M, Jabbar A, Jenkins DJ, Manfredi MT, Mirhendi H, M’rad S, Rostami-Nejad M, Oudni-M’rad M, Pierangeli NB, Ponce-Gordo F, Rehbein S, Sharbatkhori M, Kia EB, Simsek S, Soriano SV, Sprong H, Šnábel V, Umhang G, Varcasia A and Saarma U (2018a) Distinguishing *Echinococcus granulosus* sensu stricto genotypes G1 and G3 with confidence: A practical guide. *Infection Genetics and Evolution* 64, 178–184. 10.1016/j.meegid.2018.06.026. 29936039

[ref29] Kinkar L, Laurimäe T, Acosta-Jamett G, Andresiuk V, Balkaya I, Casulli A, Gasser RB, van der Giessen J, González LM, Haag KL, Zait H, Irshadullah M, Jabbar A, Jenkins DJ, Kia EB, Manfredi MT, Mirhendi H, M’rad S, Rostami-Nejad M, Oudni-M’rad M, Pierangeli NB, Ponce-Gordo F, Rehbein S, Sharbatkhori M, Simsek S, Soriano SV, Sprong H, Šnábel V, Umhang G, Varcasia A and Saarma U (2018b) Global phylogeography and genetic diversity of the zoonotic tapeworm *Echinococcus granulosus sensu stricto* genotype G1. *International Journal for Parasitology* 48, 729–742. 10.1016/j.ijpara.2018.03.006. 29782829

[ref30] Kinkar L, Laurimäe T, Balkaya I, Casulli A, Zait H, Irshadullah M, Sharbatkhori M, Mirhendi H, Rostami-Nejad M, Ponce-Gordo F, Rehbein S, Kia EB, Simsek S, Šnábel V, Umhang G, Varcasia A and Saarma U (2018c) Genetic diversity and phylogeography of the elusive, but epidemiologically important *Echinococcus granulosus sensu stricto* genotype G3. *Parasitology* 145, 1613–1622. 10.1017/S0031182018000549. 29661261

[ref31] Kinkar L, Laurimäe T, Sharbatkhori M, Mirhendi H, Kia EB, Ponce-Gordo F, Andresiuk V, Simsek S, Lavikainen A, Irshadullah M, Umhang G, Oudni-m’rad M, Acosta-Jamett G, Rehbein S and Saarma U (2017) New mitogenome and nuclear evidence on the phylogeny and taxonomy of the highly zoonotic tapeworm *Echinococcus granulosus sensu stricto*. *Infection Genetics and Evolution* 52, 52–58. 10.1016/j.meegid.2017.04.023. 28456662

[ref32] Kinkar L, Laurimäe T, Simsek S, Balkaya I, Casulli A, Manfredi MT, Ponce-Gordo F, Varcasia A, Lavikainen A, Gonzále LM, Rehbein S, Giessen JVD, Sprong H and Saarma U (2016) High-resolution phylogeography of zoonotic tapeworm *Echinococcus granulosus sensu stricto* genotype G1 with an emphasis on its distribution in Turkey, Italy and Spain. *Parasitology* 143, 1790–1801. 10.1017/S0031182016001530. 27572265

[ref33] Knapp J, Nakao M, Yanagida T, Okamoto M, Saarma U, Lavikainen A and Ito A (2011) Phylogenetic relationships within *Echinococcus* and *Taenia* tapeworms (Cestoda: Taeniidae): An inference from nuclear protein-coding genes. *Molecular Phylogenetics and Evolution* 61, 628–638. 10.1016/j.ympev.2011.07.022. 21907295

[ref34] Korhonen PK, Kinkar L, Young ND, Cai H, Lightowlers MW, Gauci C, Jabbar A, Chang BCH, Wang T, Hofmann A, Koehler AV, Li J, Li J, Wang D, Yin J, Yang H, Jenkins DJ, Saarma U, Laurimäe T, Rostami-Nejad M, Irshadullah M, Mirhendi H, Sharbatkhori M, Ponce-Gordo F, Simsek S, Casulli A, Zait H, Atoyan H, de la Rue ML, Romig T, Wassermann M, Aghayan SA, Hasmik G, Yang B and Gasser RB (2022) Chromosome-scale *Echinococcus granulosus* (genotype G1) genome reveals the Eg95 gene family and conservation of the EG95-vaccine molecule. *Communications Biology* 5, 199. 10.1038/s42003-022-03125-1. PMC8894454 35241789

[ref35] Lanfear R, Calcott B, Ho SY and Guindon S (2012) PartitionFinder: Combined selection of partitioning schemes and substitution models for phylogenetic analyses. *Molecular Biology and Evolution* 29, 1695–1701. 10.1093/molbev/mss020. 22319168

[ref36] Lanfear R, Frandsen PB, Wright AM, Senfeld T and Calcott B (2016) PartitionFinder 2: New methods for selecting partitioned models of evolution for molecular and morphological phylogenetic analyses. *Molecular Biology and Evolution* 34, 772–773. 10.1093/molbev/msw260. 28013191

[ref37] Laurimäe T, Kinkar L, Andresiuk V, Haag KL, Ponce-Gordo F, Acosta-Jamett G, Garate T, Gonzalez LM and Saarma U (2016) Genetic diversity and phylogeography of highly zoonotic Echinococcus granulosus genotype G1 in the Americas (Argentina, Brazil, Chile and Mexico) based on 8279 bp of mtDNA. *Infection Genetics and Evolution* 45, 290–296. 10.1016/j.meegid.2016.09.015. 27659214

[ref38] Laurimäe T, Kinkar L, Romig T, Omer RA, Casulli A, Umhang G, Gasser RB, Jabbar A, Sharbatkhori M, Mirhendi H, Ponce-Gordo F, Lazzarini LE, Soriano SV, Varcasia A, Rostami Nejad M, Andresiuk V, Maravilla P, Gonzalez LM, Dybicz M, Gawor J, Šarkunas M, Šnabel V, Kuzmina T and Saarma U (2018) The benefits of analysing complete mitochondrial genomes: Deep insights into the phylogeny and population structure of *Echinococcus granulosus* sensu lato genotypes G6 and G7. *Infection Genetics and Evolution* 64, 85–94. 10.1016/j.meegid.2018.06.016. 29906638

[ref39] Lemey P, Rambaut A, Drummond AJ and Suchard MA (2009) Bayesian phylogeography finds its roots. *PLOS Computational Biology* 5, e1000520. 10.1371/journal.pcbi.1000520. PMC2740835 19779555

[ref40] Lymbery AJ (2017) Phylogenetic pattern, evolutionary processes and species delimitation in the genus Echinococcus. *Advances in Parasitology* 95, 111–145. 10.1016/bs.apar.2016.07.002. 28131362

[ref41] Maksimov P, Bergmann H, Wassermann M, Romig T, Gottstein B, Casulli A and Conraths FJ (2020) Species detection within the Echinococcus granulosus sensu lato complex by novel probe-based real-time PCRs. *Pathogens* 9, 791. 10.3390/pathogens9100791. PMC7599986 32993077

[ref42] Manukyan AH, Danielyan RR, Gevorgyan KM, Sahakyan GF, Vanyan AV, Andreasyan DM and Melik-andreasyan GG (2024) Human Echinococcosis in Armenia: An Old and Neglected Challenge. *Medical Science of Armenia* 64, 80–88. 10.54503/0514-7484-2024-64.2-80.

[ref43] Ok ÜZ, Kilimcioglu AA and Özkol M (2020) Türkiye’de İnsanlarda Kistik Ekinokokkoz (Cystic Echinococcosis in Humans in Turkey). *Mikrobiyoloji Bülteni* 54, 510–522. 10.5578/mb.69712. 32755525

[ref44] Orsten S, Boufana B, Ciftci T, Akinci D, Karaagaoglu E, Ozkuyumcu C, Casulli A and Akhan O (2018) Human cystic echinococcosis in Turkey: A preliminary study on DNA polymorphisms of hydatid cysts removed from confirmed patients. *Parasitology Research* 117, 1257–1263. 10.1007/s00436-018-5807-9. 29476338

[ref45] Rambaut A (2014) Figtree, a graphical viewer of phylogenetic trees. http://tree.bio.ed.ac.uk/software/figtree (accessed 20 December 2024).

[ref46] Rambaut A, Suchard MA, Xie D and Drummond AJ (2014) Tracer v1.6. http://tree.bio.ed.ac.uk/software/tracer/ (accessed 20 December 2024).

[ref47] Romig T, Deplazes P, Jenkins D, Giraudoux P, Massolo A, Craig PS, Wassermann M, Takahashi K and de la Rue M (2017) Ecology and life cycle patterns of *Echinococcus* Species. *Advances in Parasitology* 95, 213–314. 10.1016/bs.apar.2016.11.002. 28131364

[ref48] Romig T and Wassermann M (2024) Echinococcus species in wildlife. *International Journal for Parasitology: Parasites and Wildlife* 23, 100913. 10.1016/j.ijppaw.2024.100913. PMC10884515 38405672

[ref49] Rozas J, Ferrer-mata A, Sánchez-delbarrio JC, Guirao-rico S, Librado P, Ramos-onsins SE and Sánchez-gracia A (2017) DnaSP 6: DNA sequence polymorphism analysis of large datasets. *Molecular Biology and Evolution* 34, 3299–3302. 10.1093/molbev/msx248. 29029172

[ref50] Saarma U, Jõgisalu I, Moks E, Varcasia A, Lavikainen A, Oksanen A, Simsek S, Andresiuk V, Denegri G, González LM, Ferrer E, Gárate T, Rinaldi L and Maravilla P (2009) A novel phylogeny for the genus *Echinococcus*, based on nuclear data, challenges relationships based on mitochondrial evidence. *Parasitology* 136, 317–328. 10.1017/S0031182008005453. 19154654

[ref51] Saarma U, Skirnisson K, Björnsdottir TS, Laurimäe T and Kinkar L (2023) Cystic echinococcosis in Iceland: A brief history and genetic analysis of a 46-year-old *Echinococcus* isolate collected prior to the eradication of this zoonotic disease. *Parasitology* 150, 638–643. 10.1017/S0031182023000355. PMC10261677 37161714

[ref52] Selcuk MA, Celik BA, Celik F, Celik OY, Ercan K, Uslug M, Tekin AS and Simsek S (2024) A pilot study on the epidemiology, diagnosis and characterization of *Echinococcus granulosus sensu lato* in sheep, goats and dogs in Siirt province of Türkiye revealed remarkable adaptation of *Echinococcus canadensis* (G6/G7) in goats. *Veterinary Parasitology* 332, 110320. 10.1016/j.vetpar.2024.110320. 39383687

[ref53] Simsek S and Eroksuz Y (2009) Occurrence and molecular characterization of *Echinococcus granulosus* in Turkish mouflon (*Ovis gmelinii anatolica*). *Acta Tropica* 109, 167–169. 10.1016/j.actatropica.2008.10.008 19022213

[ref54] Simsek S, Kaplan M and Ozercan IF (2011) A comprehensive molecular survey of *Echinococcus granulosus* in formalin-fixed paraffin-embedded tissues in human isolates in Turkey. *Parasitology Research* 109, 411–416. 10.1007/s00436-011-2269-8. 21286751

[ref55] Šnábel V, Altintas N, D’amelio S, Nakao M, Romig T, Yolasigmaz A, Gunes K, Turk M, Busi M, Hüttner M, Ševcová D, Ito A, Altintas N and Dubinský P (2009) Cystic echinococcosis in Turkey: Genetic variability and first record of the pig strain (G7) in the country. *Parasitology Research* 105, 145. 10.1007/s00436-009-1376-2. 19234876

[ref56] Tajima F (1989) Statistical method for testing the neutral mutation hypothesis by DNA polymorphism. *Genetics* 123, 585–595. 10.1093/genetics/123.3.585 PMC1203831 2513255

[ref57] Thompson RCA (2017) Biology and systematics of Echinococcus. *Advances in Parasitology* 95, 65–109. 10.1016/bs.apar.2016.07.001. 28131366

[ref58] Umhang G, Bastien F, Cartet A, Ahmad H, van der Ark K, Berg R, Bonelli P, Davidson RK, Deplazes P, Deksne G, Gargate MJ, van der Giessen J, Jamil N, Jokelainen P, Karamon J, Mrad S, Maksimov P, Oudni-mrad M, Muchaamba G, Oksanen A, Pepe P, Poulle M-L, Rinaldi L, Samorek-pierog M, Santolamazza F, Santoro A, Santucciu C, Saarma U, Schnyder M, Villena I, Wassermann M, Casulli A and Boue F (2025) Detection of *Echinococcus spp.* and other taeniid species in lettuces and berries: Two international multicenter studies from the MEmE project. *International Journal of Food Microbiology* 430, 111059. 10.1016/j.ijfoodmicro.2025.111059. 39787752

[ref59] Utuk AE, Simsek S, Koroglu E and McManus DP (2008) Molecular genetic characterization of different isolates of *Echinococcus granulosus* in east and southeast regions of Turkey. *Acta Tropica* 107, 192–194. 10.1016/j.actatropica.2008.05.026. 18579101

[ref60] Vural G, Baca AU, Gauci CG, Bagci O, Gicik Y and Lightowlers MW (2008) Variability in the *Echinococcus granulosus* cytochrome C oxidase 1 mitochondrial gene sequence from livestock in Turkey and a re-appraisal of the G1–3 genotype cluster. *Veterinary Parasitology* 154, 347–350. 10.1016/j.vetpar.2008.03.020. 18485601

[ref61] Yılmaz A, Aydın H, Akkaş Ö, Aydın Y, Eren S and Uslu H (2025) Molecular characterization of *Echinococcus granulosus* isolated from clinical samples of patients diagnosed with hydatid Cysts in Eastern Türkiye. *Iranian Journal of Parasitology* 20, 534–543. 10.18502/ijpa.v20i4.20461. PMC13364826 42453904

[ref62] Zeder MA (2025) Out of the shadows: Reestablishing the Eastern Fertile crescent as a center of agricultural origins: Part 1. *Journal of Archaeological Research* 33, 1–56. 10.1007/s10814-024-09195-5.

[ref63] Zhao Y, Gesang D, Wan L, Li J, Qiangba G, Danzeng W, Basang Z, Renzhen N, Yin J, Gongsang Q, Cai H, Pang H, Wang D, Asan, Zhang Q, Li J and Chen W (2022) *Echinococcus spp.* and genotypes infecting humans in Tibet Autonomous Region of China: A molecular investigation with near-complete/complete mitochondrial sequences. *Parasites and Vectors* 15, 75. 10.1186/s13071-022-05199-6. PMC8898537 35248153

