# Supplementary material for: Phylogenetic analysis of zoonotic Echinococcus granulosus sensu stricto in humans and domestic animals from Armenia and Türkiye
Source: Parasitology. 2026 May 26;153(7):880–9. doi: 10.1017/S0031182026102170 (PMC13735569; doi:10.1017/S0031182026102170)
Supplement: Supplementary file 1 [file S0031182026102170sup001.pdf]

Table S1. Data for *Echinococcus granulosus sensu stricto* G1 and G3 samples from Armenia and Türkiye.

Haplotype	Isolate	Host	Organ	Country	Region	Genotype	Collection date	GenBank accession nr
A1	Arm1	sheep	lung	Armenia	Kotayk	G1	2014	PX908801
A2	Arm2	sheep	liver	Armenia	Kotayk	G1	2014	PX908802
A3	Arm3	human	lung	Armenia	Yerevan	G1	2016	PX908803
A4	Arm5	human	liver	Armenia	Ararat	G1	2016	PX908804
A5	Arm8	human	liver	Armenia	Kotayk	G1	2016	PX908805
A6	Arm11	sheep	lung/liver	Armenia	Gegharkunik	G1	2016	PX908806
A7	Arm12	cattle	liver	Armenia	Yerevan region	G1	2016	PX908807
A6	Arm13	sheep	liver	Armenia	Kotayk	G1	2014	PX908808
A8	Arm14	sheep	lung	Armenia	Kotayk	G1	2014	PX908809
A9	Arm15	sheep	lung	Armenia	Kotayk	G1	2014	PX908810
A13	Arm19	sheep	lung	Armenia	Kotayk	G1	15-May-2015	PX908811
A21	Arm29	sheep	lung/liver	Armenia	Gegharkunik	G1	2016	PX908812
A22	Arm30	human	liver	Armenia	Kotayk	G1	15.05.2015	PX908813
A23	Arm31	cattle	lung	Armenia	Yerevan region	G1	2015	PX908814
A24	Arm32	human	lung	Armenia	Ararat	G1	2016	PX908815
A25	Arm33	cattle	lung	Armenia	Shirak	G1	2015	PX908816
A26	Arm35	human	lung	Armenia	Armavir	G1	2016	PX908817
A27	Arm40	cattle	lung	Armenia	Yerevan region	G1	2015	PX908818
A28	Arm41	sheep	lung	Armenia	Ararat	G1	2015	PX908819
A29	Arm42	cattle	lung	Armenia	Yerevan region	G1	2015	PX908820
A30	Arm43	cattle	liver	Armenia	Yerevan region	G1	2015	PX908821
A32	Arm45	sheep	lung	Armenia	Kotayk	G1	2014	PX908822
A33	Arm46	sheep	lung	Armenia	Kotayk	G1	2014	PX908823
A35	Arm48	sheep	liver	Armenia	Kotayk	G1	2014	PX908824
A36	Arm49	sheep	liver	Armenia	Kotayk	G1	2014	PX908825
A37	Arm50	sheep	lung	Armenia	Kotayk	G1	2014	PX908826
T2	E3	cattle	-	Türkiye	Elazig	G1	September, 2017	PX908827
T3	E4	cattle	-	Türkiye	Elazig	G1	September, 2017	PX908828
T3	H46	sheep	-	Türkiye	Erzincan	G1	May, 2017	PX908829
T4	H1	cattle	-	Türkiye	Erzincan	G1	May, 2017	PX908830
T5	H2	cattle	-	Türkiye	Erzincan	G1	May, 2017	PX908831
T6	H5	cattle	-	Türkiye	Erzincan	G1	May, 2017	PX908832
T7	H8	cattle	-	Türkiye	Erzincan	G1	May, 2017	PX908833
T8	H9	cattle	-	Türkiye	Erzincan	G1	May, 2017	PX908834
T9	H11	sheep	-	Türkiye	Erzincan	G1	May, 2017	PX908835
T9	H56	sheep	-	Türkiye	Erzincan	G1	May, 2017	PX908836

T10	H12	cattle	-	Türkiye	Erzincan	G1	May, 2017	PX908837
T11	H14	cattle	-	Türkiye	Erzincan	G1	May, 2017	PX908838
T12	H15	cattle	-	Türkiye	Erzincan	G1	May, 2017	PX908839
T13	H20	sheep	-	Türkiye	Erzincan	G1	May, 2017	PX908840
T14	H7	cattle	-	Türkiye	Erzincan	G1	May, 2017	PX908841
T14	H24	cattle	-	Türkiye	Erzincan	G1	May, 2017	PX908842
T15	H16	sheep	-	Türkiye	Erzincan	G1	May, 2017	PX908843
T15	H57	sheep	-	Türkiye	Erzincan	G1	May, 2017	PX908844
T16	H13	cattle	-	Türkiye	Erzincan	G1	May, 2017	PX908845
T17	H32	sheep	-	Türkiye	Erzincan	G1	May, 2017	PX908846
T18	H39	sheep	-	Türkiye	Erzincan	G1	May, 2017	PX908847
T19	H22	cattle	-	Türkiye	Erzincan	G1	May, 2017	PX908848
T20	H29	cattle	-	Türkiye	Erzincan	G1	May, 2017	PX908849
T21	H34	cattle	-	Türkiye	Erzincan	G1	May, 2017	PX908850
T22	H36	cattle	-	Türkiye	Erzincan	G1	May, 2017	PX908851
T23	H38	cattle	-	Türkiye	Erzincan	G1	May, 2017	PX908852
T25	H43	sheep	-	Türkiye	Erzincan	G1	May, 2017	PX908853
T26	H41	sheep	-	Türkiye	Erzincan	G1	May, 2017	PX908854
T27	H51	sheep	-	Türkiye	Erzincan	G1	May, 2017	PX908855
T28	H48	sheep	-	Türkiye	Erzincan	G1	May, 2017	PX908856
T29	H49	sheep	-	Türkiye	Erzincan	G1	May, 2017	PX908857
T30	H50	sheep	-	Türkiye	Erzincan	G1	May, 2017	PX908858
T31	H53	sheep	-	Türkiye	Erzincan	G1	May, 2017	PX908859
T32	H55	sheep	-	Türkiye	Erzincan	G1	May, 2017	PX908860
T33	H60	sheep	-	Türkiye	Erzincan	G1	May, 2017	PX908861
T34	H42	sheep	-	Türkiye	Erzincan	G1	May, 2017	PX908862
T34	H47	sheep	-	Türkiye	Erzincan	G1	May, 2017	PX908863
T35	H25	sheep	-	Türkiye	Erzincan	G1	May, 2017	PX908864
T35	H59	sheep	-	Türkiye	Erzincan	G1	May, 2017	PX908865
T36	T1	human	-	Türkiye	Elazig	G1	September, 2017	PX908866
T37	T2	human	-	Türkiye	Elazig	G1	September, 2017	PX908867
T38	T4	human	-	Türkiye	Elazig	G1	September, 2017	PX908868
T40	T10	human	-	Türkiye	Elazig	G1	September, 2017	PX908869
A10	Arm16	human	liver	Armenia	Lori	G3	2015	
A11	Arm17	human	lung/liver	Armenia	Lori	G3	2015	
A12	Arm18	human	liver	Armenia	Shirak	G3	2015	
A14	Arm22	human	lung	Armenia	Aragatsotn	G3	2016	
A15	Arm23	human	lung	Armenia	Yerevan	G3	2016	
A16	Arm24	cattle	lung	Armenia	Yerevan region	G3	2016	
A17	Arm25	cattle	liver	Armenia	Yerevan region	G3	2016	
A18	Arm26	sheep	lung	Armenia	Gegharkunik	G3	15.05.2015	
A19	Arm27	sheep	lung	Armenia	Kotayk	G3	15.05.2015	

A20	Arm28	sheep	-	Armenia	-	G3	2016	
A31	Arm44	sheep	lung	Armenia	Kotayk	G3	2014	
A34	Arm47	sheep	lung	Armenia	Kotayk	G3	2014	
T1	E2	cattle	-	Türkiye	Elazig	G3	September, 2017	
T1	H44	sheep	-	Türkiye	Erzincan	G3	May, 2017	
T24	H37	sheep	-	Türkiye	Erzincan	G3	May, 2017	
T39	T6	human	-	Türkiye	Elazig	G3	September, 2017	
