# Supplementary material for: Phylogenetic analysis of zoonotic Echinococcus granulosus sensu stricto in humans and domestic animals from Armenia and Türkiye
Source: Parasitology. 2026 May 26;153(7):880–9. doi: 10.1017/S0031182026102170 (PMC13735569; doi:10.1017/S0031182026102170)
Supplement: Supplementary file 2 [file S0031182026102170sup002.pdf]

Table S2. Primers used for PCR and sequencing of the mitochondrial genome of *Echinococcus granulosus sensu stricto* genotypes G1 and G3. Positions are according to the complete mitochondrial genome sequence (17,675 bp) of genotype G1 (MK774655.1 in the GenBank database; Kinkar et al. 2019).

Primer name	Primer sequence	Positions	Size
Ef1	TCGTTTTACACGCGATTGAACT	15840-15861	
Er1	ACCTGCTATGCAGCCCTATT	17063-17082	1243 bp
E2fn	GATGCTGTTAACTTCAAGAAATG	16950-16972	
E2r2	CTCAAAGCATTCAAACGC	294-311	1037 bp
E3fn	GTTGATTCGTGTTAATTTTTTGGAG	114-138	
E3rn	GAAAACATAGCAAACAACAACCC	814-836	723 bp
E4f2	GTGATCCTATTTTATTTCAAC	677-697	
E4rn	GCTACCTTTGCACAGTCAATATAC	2181-2204	1528 bp
E5fn	GGTACCTAGTTTTTGTTATATTGT	1954-1977	
E5rn	GAATCGCTCACTGCCAAACCA	3055-3075	1122 bp
E6f	TAAGGGTGATGCAATTTGAG	2830-2849	
E6r	ACAACCATCTACAGCACGAA	4054-4073	1244 bp
E7fn	GATGCTGTTCCTGGTCGTCTTAATC	3865-3899	
E7rn	CAATCAACTTCAACAACATAAACC	5351-5374	1510 bp
**S7f***	GTGTCGGTGTTTAGTCCTAATAG	4295-4317	**sequencing**
E8fn	GCTTATGTTACGGCCATAAGA	5075-5095	
E8rn	TGCTTAGTAAAAAACACCCCA	6123-6143	1069 bp
E10f	GATTACTGTTACTGGTTTTCA	11228-11248	
E10r	CAACTTAAAAACAAGCATCATCA	12672-12694	1467 bp
E11f	TTTTATGCTATTCTTCGGTGTA	12437-12458	
E11r	CAAAAACACCTCATTAAACCAC	14194-14215	1779 bp
**S11f***	GGTGCTAATTTTAGGTTTGTATAGACA	13102-13128	**sequencing**
E12f	TTGTGGTGTTTTTATGATG	13838-13856	
E12r	CACAGACGATAACCCAGA	15120-15137	1300 bp
E13f	CGGGTCTTTTATTTTGATGTTG	14919-14940	
E13r	GATCCAAAAGCACATCGA	16423-16440	1522 bp

*Primer used only for sequencing (as an additional primer)
